# Mismatch-Induced Toehold-Free Strand Displacement Used to Control a DNA Nanodevice

**DOI:** 10.1021/acssynbio.5c00107

**Published:** 2025-05-28

**Authors:** Hannah Talbot, Arun Richard Chandrasekaran

**Affiliations:** † The RNA Institute, University at Albany, State University of New York, Albany, New York 12222, United States; ‡ Department of Biological Sciences, University at Albany, State University of New York, Albany, New York 12222, United States; § Department of Nanoscale Science and Engineering, University at Albany, State University of New York, Albany, New York 12222, United States

**Keywords:** DNA nanotechnology, DNA devices, paranemic crossover DNA, strand displacement, DNA nanostructures

## Abstract

Dynamic DNA structures are controlled through toehold-based strand displacement, a method in which a DNA or RNA strand can bind to a single-stranded extension, cause branch migration, and result in the displacement of previously bound DNA. Here, we developed a toehold-free strand displacement method utilizing mismatched base pairs and stability differences between DNA complexes to control the reconfiguration of DNA nanostructures. We demonstrate this method using simple DNA duplexes and apply the strategy to reconfigure a paranemic crossover (PX) DNA based nanodevice into its topoisomer juxtaposed (JX) DNA. While the mismatch-induced toehold-free strand displacement was efficient in a simple double-stranded DNA model, the efficiency of strand displacement was lower in complex nanostructures. Increasing the number of mismatches increased the efficiency of the PX-JX conversion, and the process could be further controlled by tuning the number of mismatches. This device can be useful in stimuli-responsive mechanisms that have applications in biosensing, drug delivery, and molecular computation.

## Graphical abstract

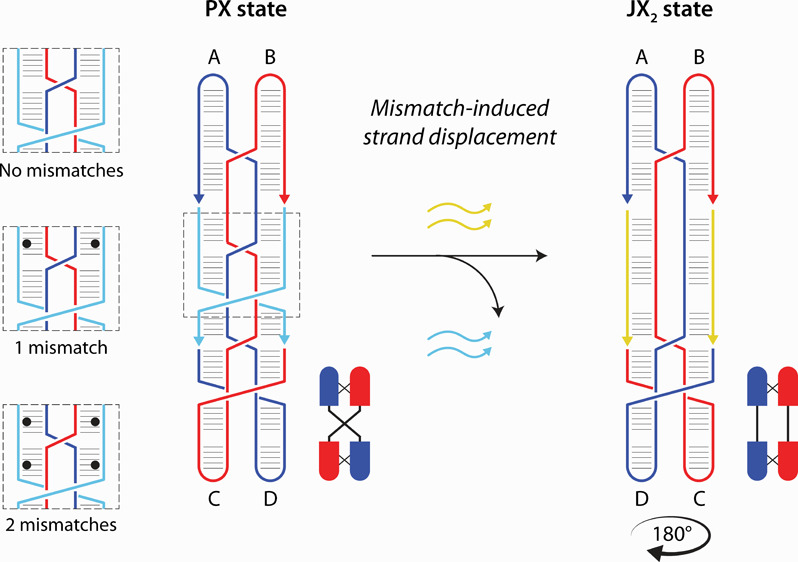

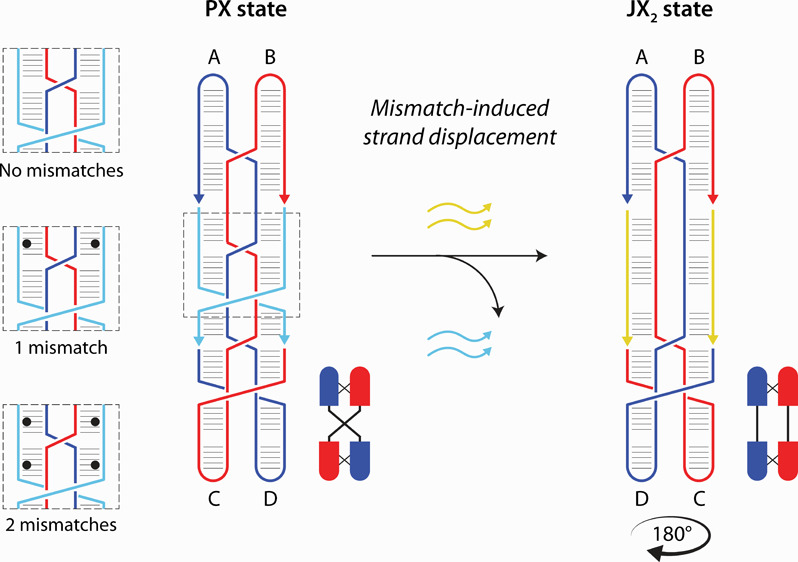

A key strategy in the reconfiguration of dynamic DNA devices is the strand displacement process, typically initiated by a single-stranded toehold.^1^ Toehold-mediated strand displacement has been used in several applications including molecular computation,^2,3^ biosensing,^4,5^ and drug delivery.^6,7^ New approaches have shown additional control over the toehold-based strand displacement process through the use of caged toeholds,^8^ antitoeholds,^9^ chemical modifications^10^ and enzymatic reaction,^11^ including our previous work on controlling DNA nanostructure disassembly using a light- or ribonuclease-based toehold clipping strategy.^12^ There has also been work to develop toehold-free strand displacement based on CRISPR-Cas systems^13^ and by modulating the base pairing kinetics of competing strands.^14^ Adding to these, we previously demonstrated toehold-free strand displacement based on structure affinity rather than sequence affinity^15,16^ and the use of ribonucleases (RNases) to initiate strand displacement.^17^ Here, we present toehold-free strand displacement reactions initiated by the presence of mismatches in DNA structures and demonstrate its use in the operation of a DNA nanodevice. Previous works have demonstrated the varying kinetics and effect on branch migration process due to mismatches on toehold sequences but not in the actual structures.^18–22^

We first tested strand displacement with mismatches in a double helical context. We used three different duplexes, each consisting of one common strand (strand X) and one complementary strand containing 0, 1, or 2 mismatches (strands Y, Y_1_, and Y_2_, respectively) that together form a 16 base pair duplex (Figure 1a and Table S1). We analyzed the duplexes using nondenaturing polyacrylamide gel electrophoresis (PAGE) and confirmed that the mismatches did not prevent duplex formation (Figure 1b). Introduction of mismatches reduced the melting temperature from ∼70 °C for the duplex with no mismatches to 65.5 and 56.5 °C for the duplex containing one and two mismatches, respectively (Figure 1c), but did not affect the stability of the complexes in solution as seen by nondenaturing PAGE.

**Figure 1:**
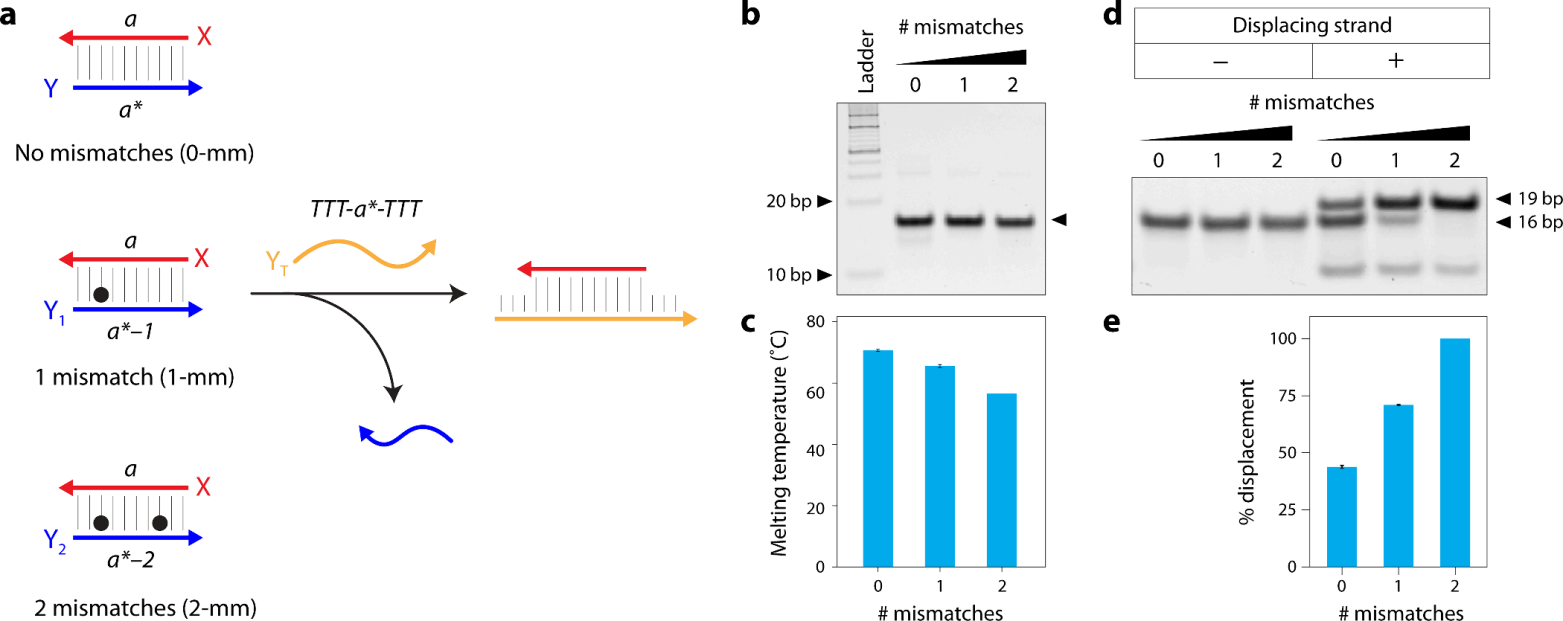
Mismatch-induced strand displacement in a duplex model. (a) Schematic of duplexes with no mismatches (duplex XY) and with 1 or 2 mismatches (duplexes XY_1_ and XY_2_, respectively). The addition of a fully complementary strand displaces the mismatch-containing strand. The displacing strand contains three Ts at each terminus, to allow separation by gel electrophoresis. (b) Nondenaturing gel showing the assembly of duplexes. (c) Melting temperatures of duplexes tested here. (d) Nondenaturing gel showing the transition from the initial duplex (lower band) to product duplex (higher band) after displacement. (e) Quantified results comparing the efficiency of displacement at room temperature (20 °C) for each tested duplex.

Next, we assembled the three duplexes and incubated them with a displacing strand (Y_T_) that is fully complementary to strand X but with three additional thymines on either end to distinguish the product duplex from the initial complex on a gel (Figure 1a). This strand forms the same number of base pairs with strand X as strand Y and has a higher affinity to strand X compared to strand Y containing mismatches, thus displacing strands Y_1_ or Y_2_ from duplexes XY_1_ and XY_2_, respectively (Figure 1d and Figure S1). With the addition of the displacing strand, an upper band appeared in the gels, indicating the occurrence of strand displacement and the formation of duplex XY_T_. We quantified the band corresponding to the product duplex and found that the displacement efficiency increased with an increasing number of mismatches (Figure 1e). We also observed this band when the displacing strand was added to the duplex containing no mismatches, likely due to strand X forming both the initial duplex XY and the product duplex XY_T_ in equal amounts (with strand Y and Y_T_, respectively). To evaluate this further, we annealed complexes containing strand X and different ratios of strands Y and Y_T_. In the presence of equal ratios of strands Y and Y_T_, we observed the formation of complexes XY and XY_T_ in almost equal proportions (Figure S2).

To demonstrate mismatch-induced strand displacement in a DNA nanotechnology context, we chose the PX-JX_2_ device.^23^ In the original version of the device, a paranemic crossover (PX) DNA state is converted to a juxtaposed DNA state that lacks two crossovers (JX_2_) through the process of toehold-mediated strand displacement (Figure 2a and Figure S3).^23^ The PX state of the device consists of two double helical domains placed side-by-side and connected by five crossovers. Two DNA set strands hold the device in the PX state and contain toeholds that activate the operation of the device. Addition of unset strands removes the set strands, resulting in a naked frame intermediate. Following this, addition of a pair of JX_2_ set strands reconfigures the frame into the JX_2_ state, causing a 180° rotation of one end of the structure relative to the other (Figure 2b). Modifications to the device have previously allowed the operation of the device using RNA cover strands,^8^ the same sequences of set strands,^24^ and our own recent work used a combination of ribonuclease and DNA strands.^17^

**Figure 2:**
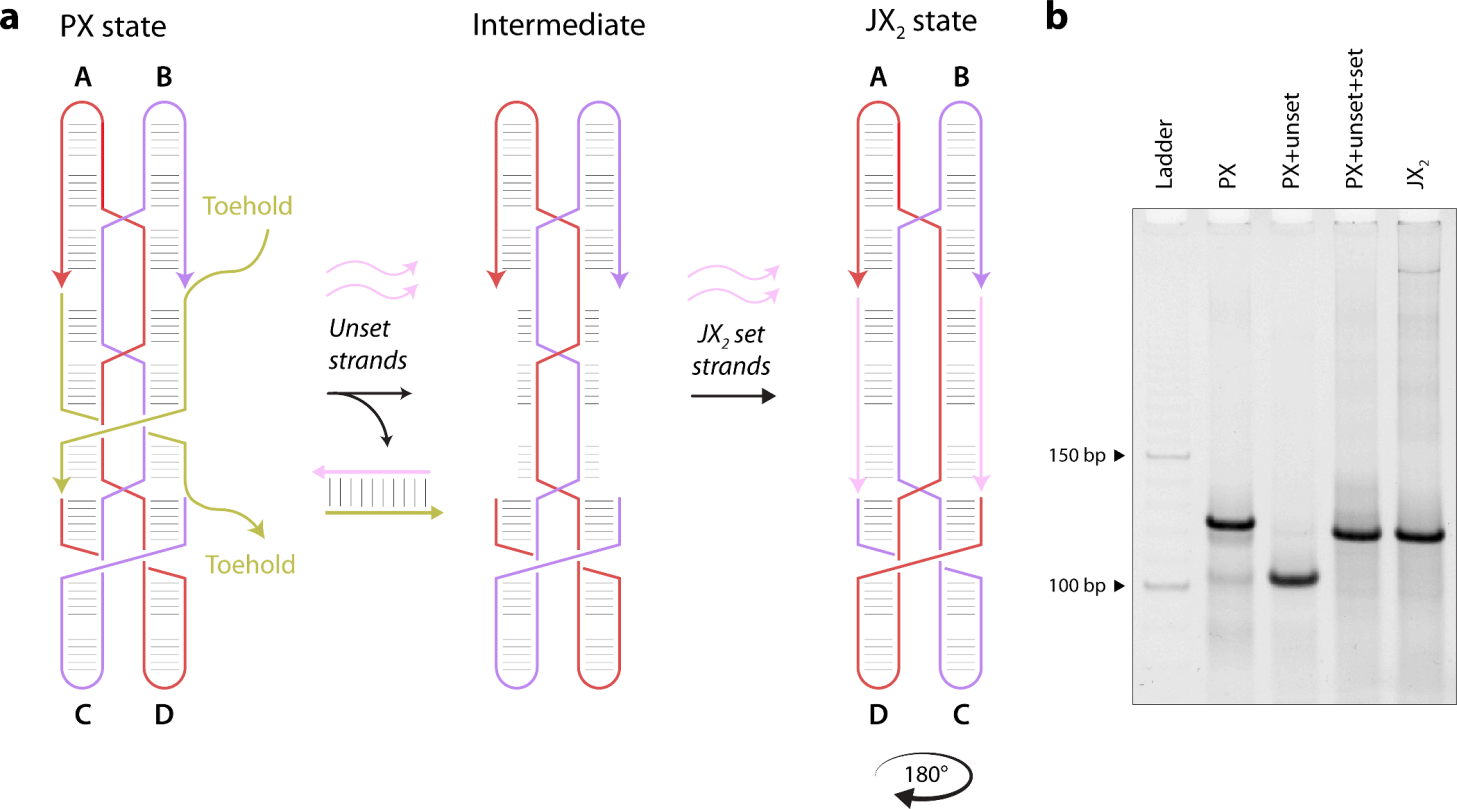
DNA rotary device controlled by toehold-based strand displacement. (a) Schematic of the DNA rotary device showing reconfiguration of the device from the paranemic crossover (PX) state to the juxtaposed crossover (JX_2_) state, which lacks two crossovers. Unset strands remove the PX set strands via toehold based strand displacement. (b) Nondenaturing gel showing reconfiguration of the device from PX to the JX_2_ state.

To test mismatch-induced strand displacement, we made two changes to the original design of the PX state. We removed the toeholds and designed set strands that each contained one or two mismatches (Figure 3a, Figure S4, and Table S2). To confirm that the introduction of mismatches did not affect the assembly of the PX state, we annealed the structures with 0, 1, or 2 pairs of mismatches and tested the assembly on a nondenaturing PAGE (Figure 3b). Structures with the mismatches were shown as a single band on the gel, indicating proper assembly and that the structure tolerates one or two mismatches per set strand. We also performed a thermal melting analysis to compare the stability of the assembled PX state containing different number of mismatches (Figure 3c). We observed that the structure without mismatches was the most thermally stable with a melting temperature of 60 °C. Introduction of mismatches reduced the melting temperature by ∼9 and ∼19 °C for the one and two pairs of mismatches, respectively.

**Figure 3:**
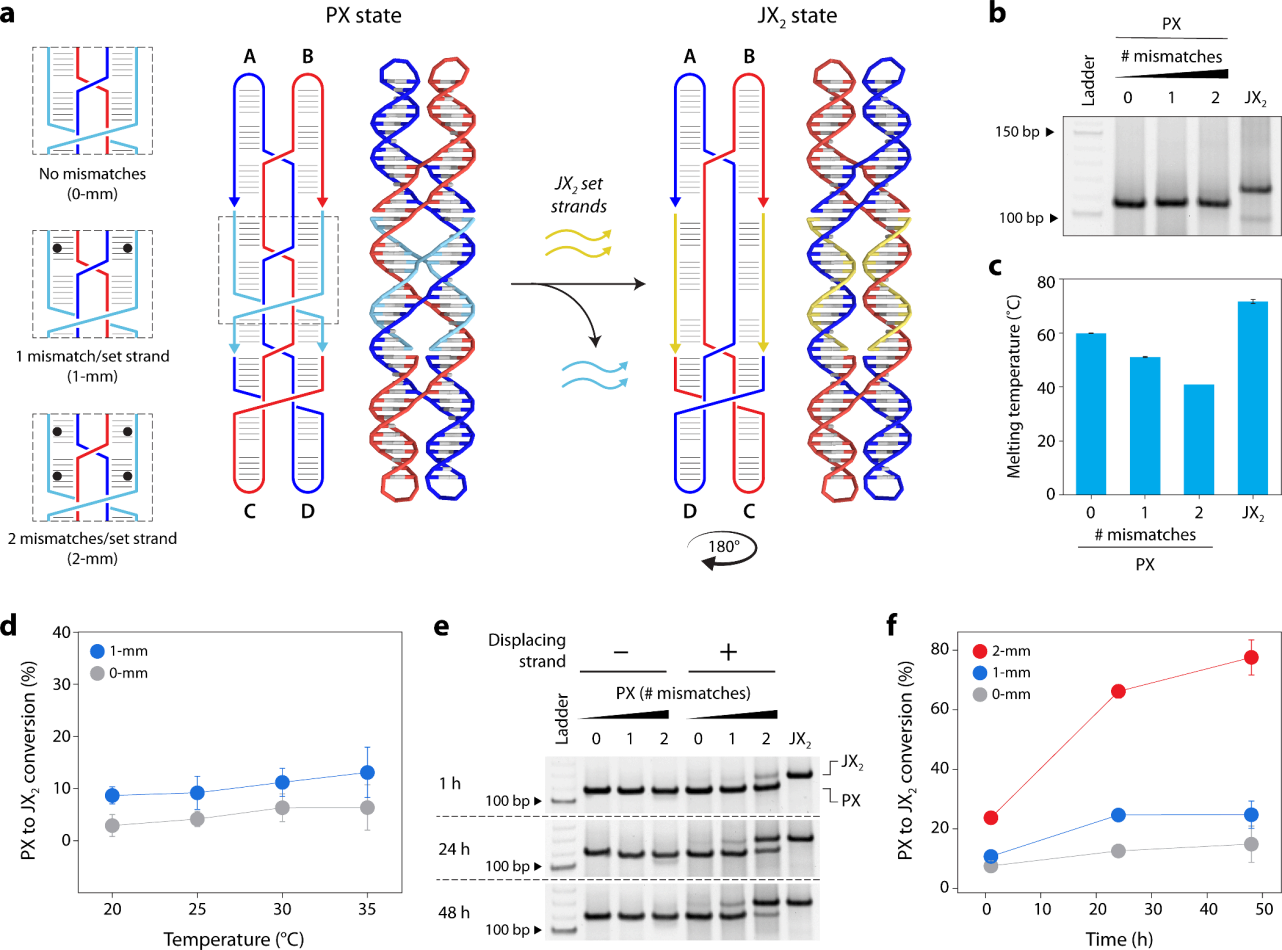
Mismatch-induced toehold-free displacement to reconfigure a DNA rotary device. (a) Design and operation of the PX DNA device containing 0 to 2 mismatches in each set strand. (b) Assembly of PX DNA with mismatches confirmed by nondenaturing PAGE. (c) Melting temperatures of the PX and JX_2_ motifs. (d) Efficiency of mismatch-induced displacement performed at different temperatures. PX motif with 2 mismatches is not stable at temperatures above 25 °C and is not included here. (e) Nondenaturing PAGE of mismatch-induced displacement for different incubation times at 25 °C and (f) efficiency of displacement quantified from gel results shown in panel (e). Data represent the mean and error propagated from standard deviations of at least three experimental replicates.

Next, we tested the efficiency of mismatch-induced displacement in each of the PX motifs. We first tested the effect of temperature on the efficiency of the displacement by incubating the PX state of the device with JX_2_ set strands (1:4 molar ratio) at 20, 25, 30, and 35 °C for 1 h. We analyzed the samples using nondenaturing PAGE and quantified the band corresponding to the JX_2_ state. We observed higher displacement at higher temperatures for the PX motif containing 1 mismatch per set strand (Figure 3d and Figure S5). We observed that the structure without any mismatches was also reconfigured at higher temperatures with the addition of JX_2_ set strands, possibly due to the higher thermal stability of the JX_2_ state of the device (Figure 3c). For the PX motif with 2 mismatches, the set strands did not stay in the structure when the temperature was increased above 25 °C (Figure S5). This could be a result of the lower thermal stability by the introduction of two mismatches, as observed with the ∼14 °C decrease in melting temperature in a duplex context and 19 °C reduction in melting temperature of the PX containing 2 mismatches. While the structure remains intact when stored at lower temperatures, the set strands containing 2 mismatches did not stay bound to the intermediate when incubated at 25 and 30 °C. Furthermore, this effect was more pronounced when both the set strands contain mismatches, when compared to PX complexes constructed using only one set strand with mismatches (with the other being a full match) (Figure S6). Thus, the presence of mismatches might cause local structural alterations^25^ that affect the robustness of the structure at higher temperatures. Other factors that affect this stability include the position of the mismatches, the length of the set strands used and the strength of base stacks^26^ across the nicked sites. We note that this structural effect is specific to this PX device built using set strands (and thus contains 4 nicks in the complex). The effect of mismatches on the assembly and stability of PX DNA in general is yet unknown. We then tested different incubation times at 25 °C to avoid affecting the structure. We added the JX_2_ set strands to each PX motif (1:4 ratio) and incubated the samples at 25 °C for up to 48 h (Figure 3e and Figure S7). We observed that displacement efficiency increased with time, with ∼24% displacement after 1 h and ∼78% displacement at 48 h for the structure with 2 mismatches (Figure 3f). We saw minimal displacement of the structure without any mismatches over this time period.

Overall, our work shows toehold-free strand displacement in DNA complexes and its use in the reconfiguration of DNA devices. Adding to existing techniques based on an external toehold sequence, our strategy provides an alternate route to controlling DNA devices by an inherent sequence choice that contains mismatches. A direct comparison of our mismatch-induced displacement strategy to toehold-based strategies is challenging since toehold-based strand displacement involves two steps: (1) the removal of the toehold-containing strand from the PX device to cause the formation of the naked intermediate and (2) binding of the set strands to reconfigure the intermediate to the JX_2_ device. However, mismatch-induced displacement is a one-step process where the JX_2_ set strands both remove the incumbent strands and also replace them to form the JX_2_ device. In the context of strand displacement, mismatches have been used mainly in toeholds to control the rates of displacement. The location of mismatches within the initial duplex has also been shown to contribute to toehold-initiated displacement rates.^22^ A similar extensive study on mismatch placement and its direct result on strand displacement as in our strategy would provide more information to achieve single base resolution to operate such DNA devices. Further understanding on how mismatch affects displacement in RNA:DNA duplexes^27^ would allow us to use RNA set strands to control the reconfiguration of DNA devices, similar to prior work that uses ribonucleases to control RNA:DNA duplexes to achieve PX-JX_2_ reconfiguration.

While our work shows strand displacement in the presence of 1 or 2 mismatches per set strand, it should be possible to tune the position of the mismatch and the sequence to elicit this response by a single mismatch. The response of such a biomolecular device to single or double mismatches could potentially be controlled by using biological nucleic acids such as a family of microRNAs that differ by only a few nucleotides. Furthermore, through the incorporation of light-responsive linkers such as azobenzene, base pairing at specific regions can be modulated by UV exposure (instead of mismatches), allowing reversibility of this strand displacement system. This strategy can also be combined with DNA-based assembly lines where PX-JX_2_ devices have been used to select and move nanoparticle cargos.^28^ Combined with our previous work that showed successful operation of the device in biofluids such as serum and urine^17^ and enhanced biostability of PX DNA,^29^ this mismatch-induced strand displacement could be used in biosensing devices and drug delivery platforms with tunable biostability.

## Supplementary Material



## Notes

Experimental procedures, additional results and DNA sequences used (PDF)
