# Supplementary material for: Mismatch-Induced Toehold-Free Strand Displacement Used to Control a DNA Nanodevice
Source: ACS Synth Biol. 2025 May 28;14(6):1931–5. doi: 10.1021/acssynbio.5c00107 (PMC12186677; doi:10.1021/acssynbio.5c00107)
Supplement: Supplementary file 1 [file sb5c00107_si_001.pdf]

Supporting information

Mismatch-induced toehold-free strand displacement used to control a DNA nanodevice

Hannah Talbot[1,2] and Arun Richard Chandrasekaran[1,2,3,*]

[1]The RNA Institute, University at Albany, State University of New York, Albany, NY, USA.
[2]Department of Biological Sciences, University at Albany, State University of New York, Albany, NY, USA.
[3]Department of Nanoscale Science and Engineering, University at Albany, State University of New York, Albany, NY, USA.

*Correspondence: arun@albany.edu

MATERIALS AND METHODS

Preparation of DNA complexes

All DNA strands were purchased from Integrated DNA Technologies (IDT). Full sequences are listed in Table S1 and S2. To form each PX complex, DNA strands were combined in equal ratios at a final concentration of 250 nM in Tris-Acetic-EDTA buffer with Mg^{2+}, containing 40 mM Tris base (pH 8), 20 mM acetic acid, 2 mM EDTA, and 12.5 mM magnesium acetate (1× TAE-Mg^{2+}). Samples were placed in 2 L of deionized water heated to 90 °C, then placed in a Styrofoam box to cool to 20 °C over the course of 2 days. For duplexes, strands were combined in equal ratios in 1× TAE-Mg^{2+} buffer and annealed using a thermocycler from 90 °C to 20 °C over 30 minutes. Following annealing, all samples were stored at 4 °C.

Gel electrophoresis

Non-denaturing gels were prepared with different percentages of polyacrylamide (19:1 acrylamide/bisacrylamide, National Diagnostics) depending on the sample. Samples were mixed with loading dye containing bromophenol blue and glycerol prior to loading. Gels were run at a constant voltage at 4 °C with 1× TAE-Mg^{2+} running buffer. Gels were stained in 0.5× GelRed (Biotium), imaged using a Bio-Rad Gel Doc XR+ and analyzed using ImageLab software. Percent displacement was calculated as the % intensity of the band corresponding to JX_2 compared to the total intensity of the PX and JX_2 bands.

UV melting

UV melting experiments were performed on a Cary 3500 UV-Visible Spectrophotometer (Agilent) using 1.5 µM DNA concentration for the PX and JX_2 samples and 5 µM for the duplexes. Absorbance at 260 nm was recorded while samples were heated from 15 °C to 95 °C at a rate of 0.5 °C/min. This data was normalized to 0-1 and fitted to the Boltzmann curve using OriginPro. The full temperature range was used for the duplex and JX_2 fitting curves. The range was adjusted for each of the PX mismatch motifs to account for the biphasic melting behavior of PX. The ranges used were 55-65 °C, 45-55 °C and 35-45 °C for PX, PX 1-mm and PX 2-mm motifs respectively.

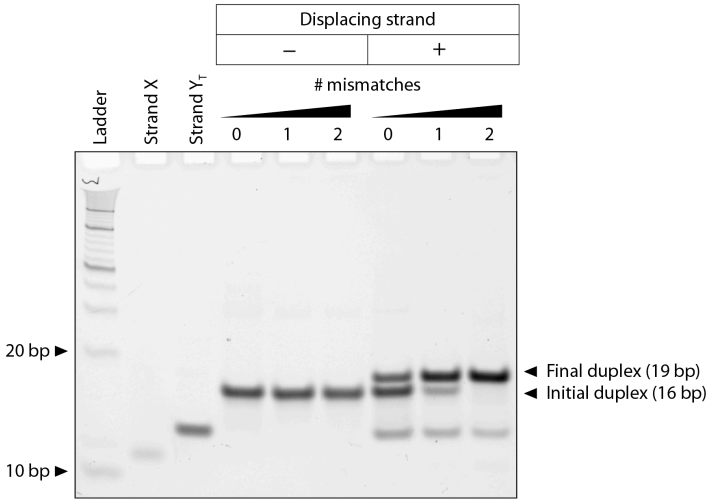

Figure S1. Non-denaturing gel showing the transition from the initial duplex (lower band) to product duplex (higher band) after displacement. Full image of gel shown in Figure 1d.

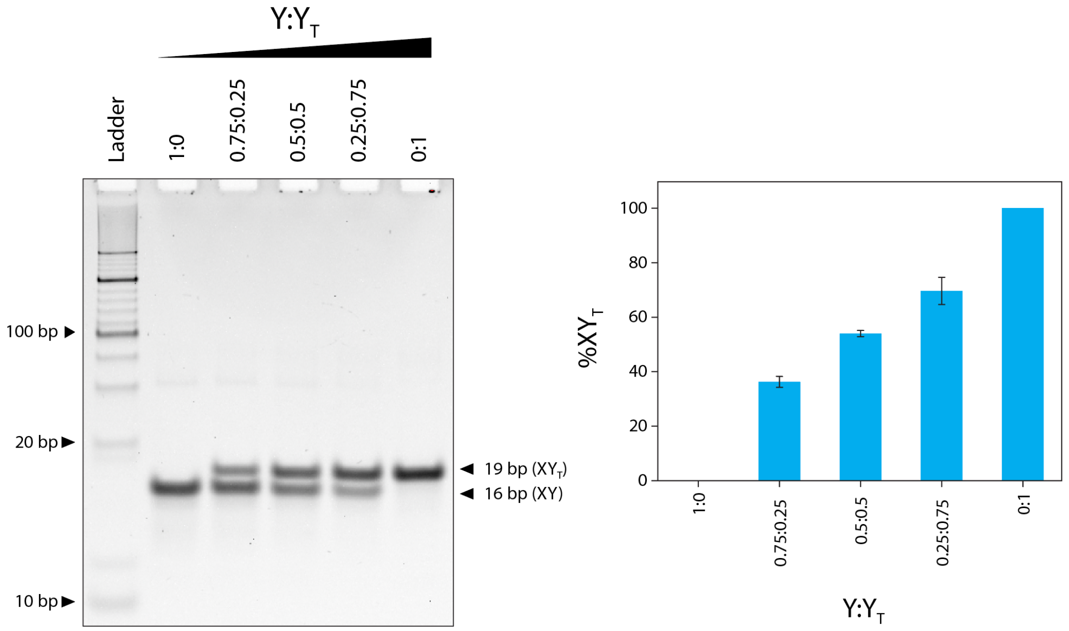

Figure S2. Binding of strand Y_T to strand X in the presence of strand Y.

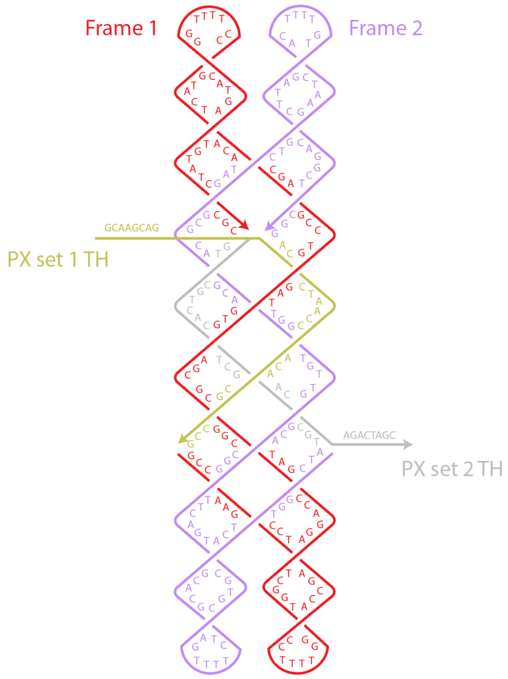

Figure S3. Sequences of the PX state with toeholds.

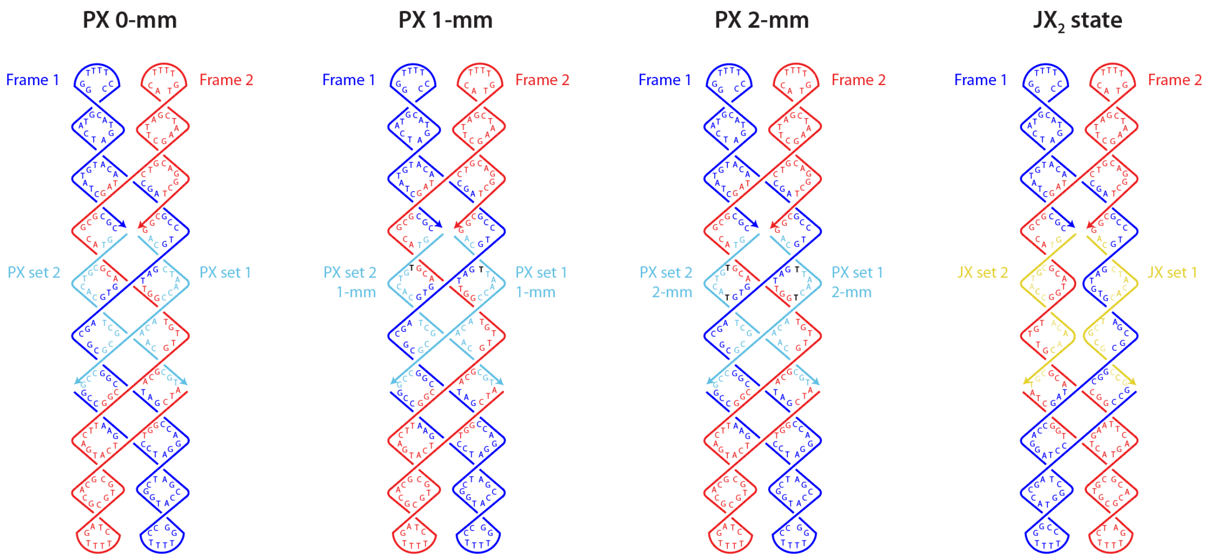

Figure S4. Sequences of the PX state with and without mismatches and the JX$_2$ state.

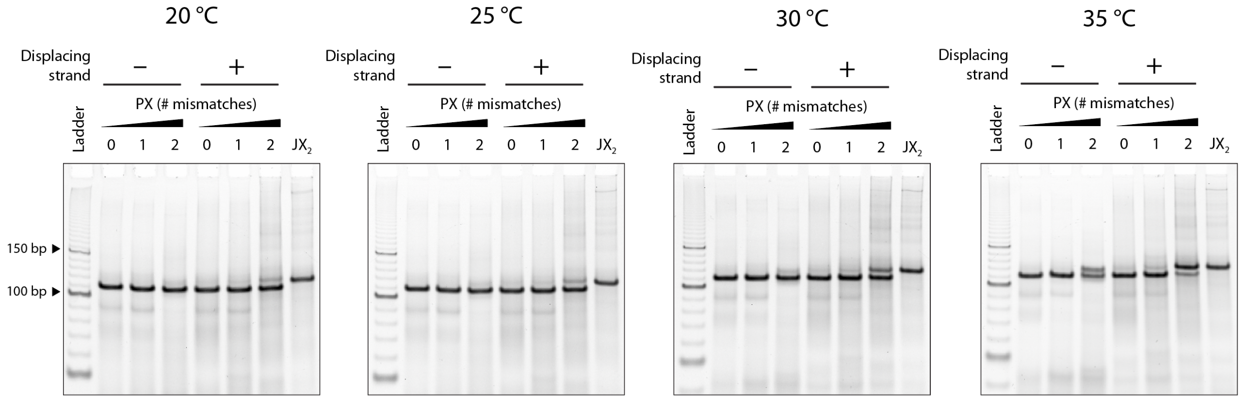

Figure S5. Non-denaturing PAGE of mismatch-induced displacement performed at different temperatures.

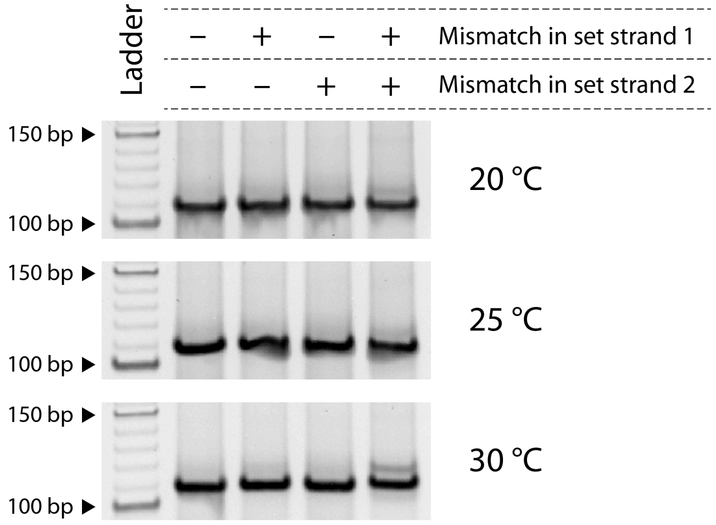

Figure S6. Non-denaturing PAGE showing the stability of the PX device containing different types of set strands when incubated at 20 °C, 25 °C and 30 °C.

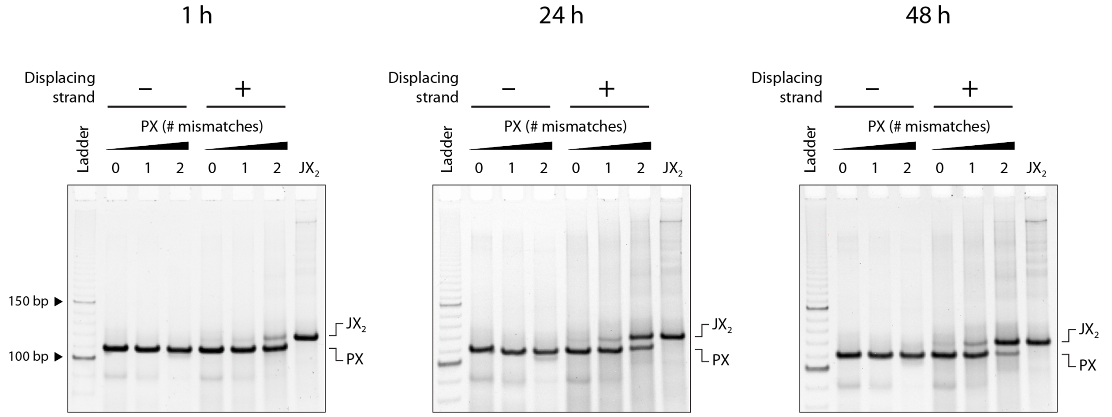

Figure S7. Non-denaturing PAGE of mismatch-induced displacement performed at different time points at 25 °C. Full images of gels shown in Figure 3e.

Strand	Sequence
X	CGGCGTGTGGTTAGGT
Y	ACCTAACCACACGCCG
Y_1	ACTTAACCACACGCCG
Y_2	ACTTAACTACACGCCG
Y_T	TTTACCTAACCACACGCCGTTT

Table S1. Duplex sequences (written 5' to 3').

Strand	Sequence
PX set 1 0-mm	ACCTAACCACACGCCG
PX set 2 0-mm	GTCGTCACTCGACCGT
PX set 1 1-mm	ACTTAACCACACGCCG
PX set 2 1-mm	GTTGTCACTCGACCGT
PX set 1 2-mm	ACTTAACTACACGCCG
PX set 2 2-mm	GTTGTCATTCGACCGT
Frame 1	GCCAAGCCTAGCCACCTTTTGGTGGCTAGGACCGATCGGCGCGA GTGTAGGTCCGAGCACATCATGCCTTTTGGCATGATGTATCCGC
Frame 2	ATCGGTTCAGCACGTCTTTTGACGTGCTGACTTGGCACGGTTGT GGTACGACGCGGATCTGGAATCACTTTTGTGATTCCAGGCTCGG
PX set 1 TH	GCAAGCAGACCTAACCACACGCCG
PX set 2 TH	GTCGTCACTCGACCGTAGACTAGC
PX unset 1 TH-comp	CGGCGTGTGGTTAGGTCTGCTTGC
PX unset 2 TH-comp	GCTAGTCTACGGTCGAGTGACGAC
JX set 1	GTCGTACCACAACCGT
JX set 2	ACCTACACTCGCGCCG

Table S2. PX and JX_2 sequences (written 5' to 3').
